# Supplementary material for: Overlapping cell population expression profiling and regulatory inference in C. elegans
Source: BMC Genomics. 2016 Feb 29;17:159. doi: 10.1186/s12864-016-2482-z (PMC4772325; doi:10.1186/s12864-016-2482-z)
Supplement: Additional file 13: — Web supplement. (DOC 21 kb) [file 12864_2016_2482_MOESM13_ESM.zip › sortWeb/clusters/hier.300.clusters/91.html]

Cluster 91 

## Cluster 91

### Expression

| cnd-1 rep. 1 | cnd-1 rep. 2 | cnd-1 rep. 3 | pha-4 rep. 1 | pha-4 rep. 2 | pha-4 rep. 3 | ceh-27 | ceh-36 | ceh-6 | F21D5.9 | mir-57 | mls-2 | pal-1 | pros-1 | ttx-3 | unc-130 | hlh-16 | irx-1 | ceh-6 (+) hlh-16 (+) | ceh-6 (+) hlh-16 (-) | ceh-6 (-) hlh-16 (+) | cnd-1 singlets | pha-4 singlets | 0 | 60 | 120 | 150 | 180 | 240 | 330 | 390 | 420 | 480 | 540 | 570 | 600 | 630 | 660 | NAME | Functional description |
| --- | --- | --- | --- | --- | --- | --- | --- | --- | --- | --- | --- | --- | --- | --- | --- | --- | --- | --- | --- | --- | --- | --- | --- | --- | --- | --- | --- | --- | --- | --- | --- | --- | --- | --- | --- | --- | --- | --- | --- |
|  |  |  |  |  |  |  |  |  |  |  |  |  |  |  |  |  |  |  |  |  |  |  |  |  |  |  |  |  |  |  |  |  |  |  |  |  |  | R12E2.17 |  |
|  |  |  |  |  |  |  |  |  |  |  |  |  |  |  |  |  |  |  |  |  |  |  |  |  |  |  |  |  |  |  |  |  |  |  |  |  |  | *arrd-13* | ARRestin Domain protein |
|  |  |  |  |  |  |  |  |  |  |  |  |  |  |  |  |  |  |  |  |  |  |  |  |  |  |  |  |  |  |  |  |  |  |  |  |  |  | R10E11.10 |  |
|  |  |  |  |  |  |  |  |  |  |  |  |  |  |  |  |  |  |  |  |  |  |  |  |  |  |  |  |  |  |  |  |  |  |  |  |  |  | *srsx-15* | Serpentine Receptor, class SX |
|  |  |  |  |  |  |  |  |  |  |  |  |  |  |  |  |  |  |  |  |  |  |  |  |  |  |  |  |  |  |  |  |  |  |  |  |  |  | *gyg-3* | GlYcoGenin like |
|  |  |  |  |  |  |  |  |  |  |  |  |  |  |  |  |  |  |  |  |  |  |  |  |  |  |  |  |  |  |  |  |  |  |  |  |  |  | M01H9.4 |  |
|  |  |  |  |  |  |  |  |  |  |  |  |  |  |  |  |  |  |  |  |  |  |  |  |  |  |  |  |  |  |  |  |  |  |  |  |  |  | C02B4.t1 |  |
|  |  |  |  |  |  |  |  |  |  |  |  |  |  |  |  |  |  |  |  |  |  |  |  |  |  |  |  |  |  |  |  |  |  |  |  |  |  | W08A12.3 |  |
|  |  |  |  |  |  |  |  |  |  |  |  |  |  |  |  |  |  |  |  |  |  |  |  |  |  |  |  |  |  |  |  |  |  |  |  |  |  | H36L18.1 |  |
|  |  |  |  |  |  |  |  |  |  |  |  |  |  |  |  |  |  |  |  |  |  |  |  |  |  |  |  |  |  |  |  |  |  |  |  |  |  | K11G9.11 |  |
|  |  |  |  |  |  |  |  |  |  |  |  |  |  |  |  |  |  |  |  |  |  |  |  |  |  |  |  |  |  |  |  |  |  |  |  |  |  | *atf-2* | ATF (cAMP-dependent transcription factor) family |
|  |  |  |  |  |  |  |  |  |  |  |  |  |  |  |  |  |  |  |  |  |  |  |  |  |  |  |  |  |  |  |  |  |  |  |  |  |  | W05E10.1 |  |
|  |  |  |  |  |  |  |  |  |  |  |  |  |  |  |  |  |  |  |  |  |  |  |  |  |  |  |  |  |  |  |  |  |  |  |  |  |  | Y105C5A.1271 |  |
|  |  |  |  |  |  |  |  |  |  |  |  |  |  |  |  |  |  |  |  |  |  |  |  |  |  |  |  |  |  |  |  |  |  |  |  |  |  | *ptr-20* | PaTched Related family |
|  |  |  |  |  |  |  |  |  |  |  |  |  |  |  |  |  |  |  |  |  |  |  |  |  |  |  |  |  |  |  |  |  |  |  |  |  |  | Y54F10AM.6 |  |
|  |  |  |  |  |  |  |  |  |  |  |  |  |  |  |  |  |  |  |  |  |  |  |  |  |  |  |  |  |  |  |  |  |  |  |  |  |  | *twk-7* | TWiK family of potassium channels |
|  |  |  |  |  |  |  |  |  |  |  |  |  |  |  |  |  |  |  |  |  |  |  |  |  |  |  |  |  |  |  |  |  |  |  |  |  |  | *hpo-36* | Hypersensitive to POre-forming toxin |
|  |  |  |  |  |  |  |  |  |  |  |  |  |  |  |  |  |  |  |  |  |  |  |  |  |  |  |  |  |  |  |  |  |  |  |  |  |  | *mec-7* | MEChanosensory abnormality |
|  |  |  |  |  |  |  |  |  |  |  |  |  |  |  |  |  |  |  |  |  |  |  |  |  |  |  |  |  |  |  |  |  |  |  |  |  |  | F45H10.5 |  |
|  |  |  |  |  |  |  |  |  |  |  |  |  |  |  |  |  |  |  |  |  |  |  |  |  |  |  |  |  |  |  |  |  |  |  |  |  |  | F58E2.5 |  |
|  |  |  |  |  |  |  |  |  |  |  |  |  |  |  |  |  |  |  |  |  |  |  |  |  |  |  |  |  |  |  |  |  |  |  |  |  |  | *ssq-1* | Sperm-Specific family, class Q |
|  |  |  |  |  |  |  |  |  |  |  |  |  |  |  |  |  |  |  |  |  |  |  |  |  |  |  |  |  |  |  |  |  |  |  |  |  |  | *lst-5* | Lateral Signaling Target |
|  |  |  |  |  |  |  |  |  |  |  |  |  |  |  |  |  |  |  |  |  |  |  |  |  |  |  |  |  |  |  |  |  |  |  |  |  |  | *rgs-9* | Regulator of G protein Signaling |
|  |  |  |  |  |  |  |  |  |  |  |  |  |  |  |  |  |  |  |  |  |  |  |  |  |  |  |  |  |  |  |  |  |  |  |  |  |  | F55G7.5 |  |
|  |  |  |  |  |  |  |  |  |  |  |  |  |  |  |  |  |  |  |  |  |  |  |  |  |  |  |  |  |  |  |  |  |  |  |  |  |  | M163.9 |  |
|  |  |  |  |  |  |  |  |  |  |  |  |  |  |  |  |  |  |  |  |  |  |  |  |  |  |  |  |  |  |  |  |  |  |  |  |  |  | F28F9.2 |  |
|  |  |  |  |  |  |  |  |  |  |  |  |  |  |  |  |  |  |  |  |  |  |  |  |  |  |  |  |  |  |  |  |  |  |  |  |  |  | E03H12.5 |  |
|  |  |  |  |  |  |  |  |  |  |  |  |  |  |  |  |  |  |  |  |  |  |  |  |  |  |  |  |  |  |  |  |  |  |  |  |  |  | Y50E8A.6 |  |
|  |  |  |  |  |  |  |  |  |  |  |  |  |  |  |  |  |  |  |  |  |  |  |  |  |  |  |  |  |  |  |  |  |  |  |  |  |  | *linc-80* | Long Intervening Non-Coding RNA |
|  |  |  |  |  |  |  |  |  |  |  |  |  |  |  |  |  |  |  |  |  |  |  |  |  |  |  |  |  |  |  |  |  |  |  |  |  |  | *fbxb-35* | F-box B protein |
|  |  |  |  |  |  |  |  |  |  |  |  |  |  |  |  |  |  |  |  |  |  |  |  |  |  |  |  |  |  |  |  |  |  |  |  |  |  | *thn-1* | THaumatiN family |
|  |  |  |  |  |  |  |  |  |  |  |  |  |  |  |  |  |  |  |  |  |  |  |  |  |  |  |  |  |  |  |  |  |  |  |  |  |  | *cpg-4* | Chondroitin ProteoGlycan |
|  |  |  |  |  |  |  |  |  |  |  |  |  |  |  |  |  |  |  |  |  |  |  |  |  |  |  |  |  |  |  |  |  |  |  |  |  |  | *sul-1* | SULfatase domain protein |

### Phenotypes enriched

none found

### Anatomy terms enriched

none found

### GO terms enriched

none found

### Expression clusters enriched

none found

### Motifs enriched

|  |  |  |  |  |  |
| --- | --- | --- | --- | --- | --- |
| **Motif** | **Logo** | **Possible orthologs** | **Number of motifs in cluster** | **Enrichment** | **FDR corrected p** |
| GM12878\_EGR1\_HudsonAlpha |  | ZC328.2 | 26 | 2.03 | 0.00068 |
| Six4\_2860 |  | ceh-32 | 6 | 12.39 | 0.00140 |
| V$RORA1\_01 |  | nhr-213 (-0.57) nhr-71 nhr-10 nhr-2 | 24 | 2.03 | 0.00280 |
| MA0545.1 |  | hlh-1 hlh-11 | 18 | 2.59 | 0.00340 |
| pTH6142 |  | nhr-213 (-0.57) nhr-69 nhr-19 nhr-2 | 11 | 4.12 | 0.00460 |
| Six1\_0935 |  | ceh-32 | 5 | 12.89 | 0.00510 |
| pTH4269 |  | nhr-86 nhr-177 nhr-2 | 18 | 2.40 | 0.00800 |
| V$GATA1\_05 |  | elt-1 (0.7) | 29 | 1.57 | 0.01000 |
| MA0035.3 |  | elt-1 (0.7) | 29 | 1.56 | 0.01100 |
| pTH1001 |  | dnj-17 | 30 | 1.50 | 0.01100 |
| pTH6327 |  | dsc-1 | 14 | 2.88 | 0.01200 |
| NR2F6\_1 |  | nhr-2 | 32 | 1.37 | 0.01500 |
| SP3\_f1 |  | klf-2 | 29 | 1.52 | 0.01700 |
| Nkx2-6\_3437 |  | ceh-24 dsc-1 | 12 | 3.14 | 0.01700 |
| pTH9326 |  | nhr-122 | 26 | 1.67 | 0.01800 |
| pTH5928 |  | ceh-34 | 17 | 2.32 | 0.01900 |
| Hr46\_SANGER\_5\_FBgn0000448 |  | nhr-213 (-0.57) nhr-118 | 18 | 2.21 | 0.01900 |
| pTH9118 |  | eor-1 egrh-3 | 27 | 1.60 | 0.02200 |
| NR2F1\_3 |  | nhr-2 | 17 | 2.28 | 0.02200 |
| pTH9073 |  | end-3 | 26 | 1.65 | 0.02200 |
| GATA5\_f1 |  | elt-1 (0.7) | 29 | 1.49 | 0.02400 |
| klu\_SOLEXA\_5\_FBgn0013469 |  | ZC328.2 | 24 | 1.74 | 0.02600 |
| HUVEC\_GATA2\_UCD |  | elt-1 (0.7) | 28 | 1.53 | 0.02700 |
| Six6\_2267 |  | ceh-34 | 4 | 11.28 | 0.03100 |
| TBX2\_f1 |  | tbx-39 | 25 | 1.66 | 0.03100 |
| pTH5561 |  | nhr-239 | 22 | 1.82 | 0.03100 |
| tj\_SANGER\_5\_FBgn0000964 |  | F45H11.6 | 30 | 1.42 | 0.03200 |
| pTH2353 |  | B0310.2 | 11 | 3.08 | 0.03300 |
| pTH5781 |  | ceh-32 | 31 | 1.37 | 0.03500 |
| pTH5437 |  | ceh-34 | 8 | 4.05 | 0.03900 |
| pTH10041 |  | ztf-29 | 31 | 1.36 | 0.03900 |
| ARNT2\_si |  | aha-1 | 26 | 1.58 | 0.04100 |
| HXB1\_f1 |  | ceh-12 | 15 | 2.33 | 0.04100 |
| Hr46\_FlyReg\_FBgn0000448 |  | nhr-213 (-0.57) | 7 | 4.56 | 0.04400 |
| pTH9108 |  | daf-12 | 9 | 3.47 | 0.04800 |
| twi\_da\_SANGER\_5\_FBgn0003900 |  | hlh-8 | 17 | 2.09 | 0.04800 |
| FLI1\_4 |  | lin-1 (0.67) | 11 | 2.90 | 0.04900 |
| MA0117.1 |  | F45H11.6 | 5 | 6.74 | 0.04900 |
| HLH27 |  | hlh-27 | 25 | 1.60 | 0.05000 |

### Correlated (and anti-correlated) transcription factors

|  |  |
| --- | --- |
| **Transcription factor** | **Correlation** |
| bar-1 | 0.85 |
| lin-39 | 0.77 |
| mab-9 | 0.74 |
| egl-46 | 0.73 |
| unc-62 | 0.72 |
| elt-1 | 0.70 |
| ceh-5 | 0.70 |
| lin-1 | 0.67 |
| hlh-32 | 0.67 |
| unc-130 | 0.67 |
| cnd-1 | 0.65 |
| F21A9.2 | 0.64 |
| lin-11 | 0.63 |
| mab-5 | 0.62 |
| F21D5.9 | 0.62 |
| hlh-13 | 0.62 |
| lst-5 | 0.62 |
| hlh-17 | 0.61 |
| ceh-13 | 0.60 |
| nhr-152 | 0.59 |
| attf-4 | 0.58 |
| ceh-20 | 0.57 |
| zag-1 | 0.57 |
| Y41D4B.26 | 0.56 |
| F26H9.2 | 0.55 |
| nhr-134 | -0.50 |
| nhr-132 | -0.50 |
| jun-1 | -0.50 |
| ceh-28 | -0.51 |
| nhr-141 | -0.51 |
| nhr-140 | -0.51 |
| nhr-105 | -0.52 |
| nhr-275 | -0.53 |
| nhr-30 | -0.53 |
| nhr-58 | -0.54 |
| dmd-9 | -0.54 |
| zip-2 | -0.56 |
| nhr-213 | -0.57 |
| nhr-50 | -0.57 |
| nhr-45 | -0.58 |
| K12H6.12 | -0.58 |
| nhr-21 | -0.58 |
| fkh-2 | -0.59 |
| nhr-116 | -0.60 |
| nhr-182 | -0.61 |
| nhr-268 | -0.62 |
| nhr-286 | -0.64 |
| nhr-84 | -0.67 |
| nhr-135 | -0.68 |
| nhr-102 | -0.69 |

### ChIP peaks enriched

none found
